# Supplementary material for: Identification of a terpene synthase arsenal using long-read sequencing and genome assembly of Aspergillus wentii
Source: BMC Genomics. 2024 Nov 26;25:1141. doi: 10.1186/s12864-024-11064-w (PMC11600568; doi:10.1186/s12864-024-11064-w)
Supplement: Supplementary file 1 — Supplementary Material 1. [file 12864_2024_11064_MOESM1_ESM.docx]

**Identification of a terpene synthase arsenal using long-read sequencing and genome assembly of *Aspergillus wentii***

Richard Olumakaiye,^1^ Christophe Corre^1,2^ & Fabrizio Alberti^1^*

﻿^1^ School of Life Sciences, University of Warwick, Coventry, CV4 7AL, UK.

^2^ Department of Chemistry, University of Warwick, Coventry, CV4 7AL, UK.

*E-mail for correspondence: F.Alberti@warwick.ac.uk.

**Supplementary Information**

**Table of Contents**

[Supplementary Figure 1: High Molecular Weight (HMW) gDNA from A. wentii CBS 141173 2](#_Toc181194037)

[Supplementary Table 1: Concentration of High Molecular Weight (HMW) A. wentii gDNA. 2](#_Toc181194038)

[Supplementary Table 2: Summary of RNA-seq analysis report of Aspergillus wentii CBS 141173 3](#_Toc181194039)

[Supplementary Figure 2: Assignment of predicted proteins in A. wentii CBS 141173 using eggnog mapper to multiple databases. 4](#_Toc181194040)

[Supplementary Figure 3. Distribution of predicted proteins in Aspergillus wentii CBS 141173 across the different COG categories. 5](#_Toc181194041)

[Supplementary Table 3: BUSCO assessment of the annotated genome and predicted proteome and transcriptome of A. wentii CBS 141173 using the Eurotiales odb10 database. 6](#_Toc181194042)

[Supplementary Figure 4. MS^2^ spectra of asperolide A. 7](#_Toc181194043)

[Supplementary Table 4: Transcriptomic hitcounts of terpene synthase genes in Aspergillus wentii CBS 141173 at two timepoints (7 and 30 days) performed in duplicate (A and B), and compared to the housekeeping gene beta-tubulin. 8](#_Toc181194044)

[Supplementary Table 5: NCBI BioProject ID submission and Accession number of A. wentii CBS 141173. 9](#_Toc181194045)


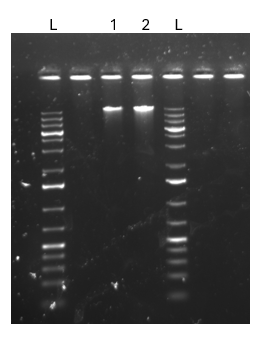


Supplementary Figure 1: High Molecular Weight (HMW) gDNA from *A. wentii* CBS 141173. Lanes L = 1kb plus ladder (Thermo Scientific). Lane 1 and 2= gDNA from *A. wentii*.

# Supplementary Table 1: Concentration of High Molecular Weight (HMW) *A. wentii* gDNA.

| Sample | | Before ethanol precipitation (ng/µL) | After ethanol precipitation  (ng/µL) |
| --- | --- | --- | --- |
| *Aspergillus wentii* CBS 141173 - 1 | | 65.4 | 481.2 |
| *Aspergillus wentii* CBS 141173 - 2 | | 60.6 | 475.5 |
|  |  |  |  |

# Supplementary Table 2: Summary of RNA-seq analysis report of *Aspergillus wentii* CBS 141173

| Sample ID | Raw PE Reads | Read pairs after deduplication and quality trimming | % Duplication Rate | Total Mapped Reads | & Total Mapped Reads | Unique Mapped Reads | % Unique Mapped Reads |
| --- | --- | --- | --- | --- | --- | --- | --- |
| Awentii7A | 14,193,289 | 12,162,221 | 12.99 | 9,407,395 | 77.35 | 9,338,336 | 76.78 |
| Awentii7B | 14,009,686 | 11,972,577 | 13.35 | 9,440,227 | 78.85 | 9,373,124 | 78.29 |
| Awentii30A | 12,697,660 | 10,745,915 | 14.07 | 8,623,884 | 80.25 | 8,575,636 | 79.80 |
| Awentii30B | 13,059,646 | 11,199,580 | 12.89 | 8,887,091 | 79.35 | 8,837,628 | 78.91 |


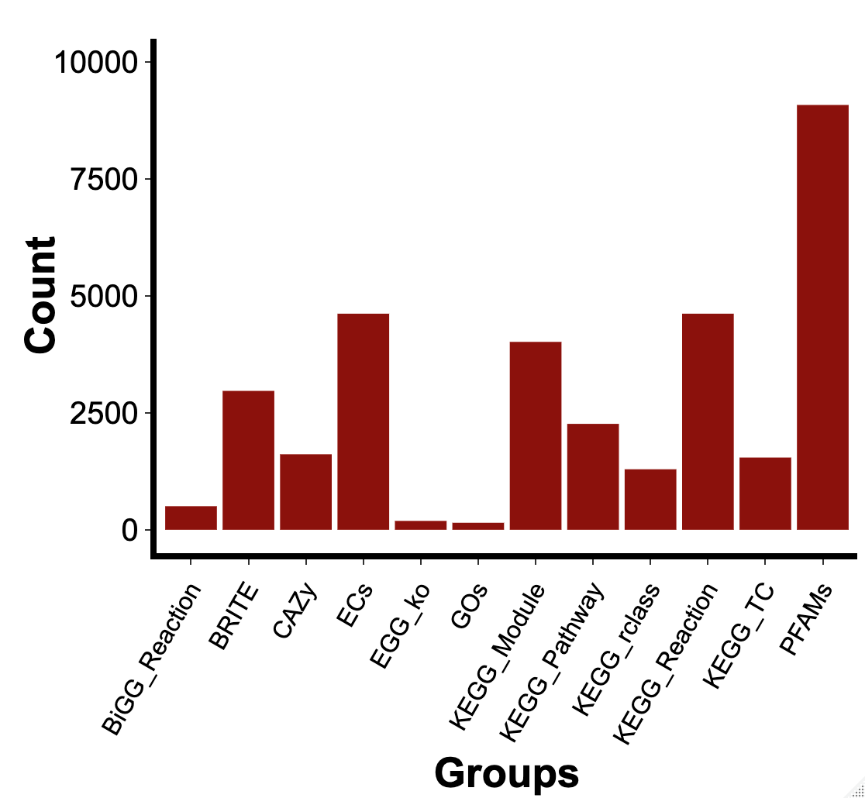


# Supplementary Figure 2: Assignment of predicted proteins in *A. wentii* CBS 141173 using eggnog mapper to multiple databases.


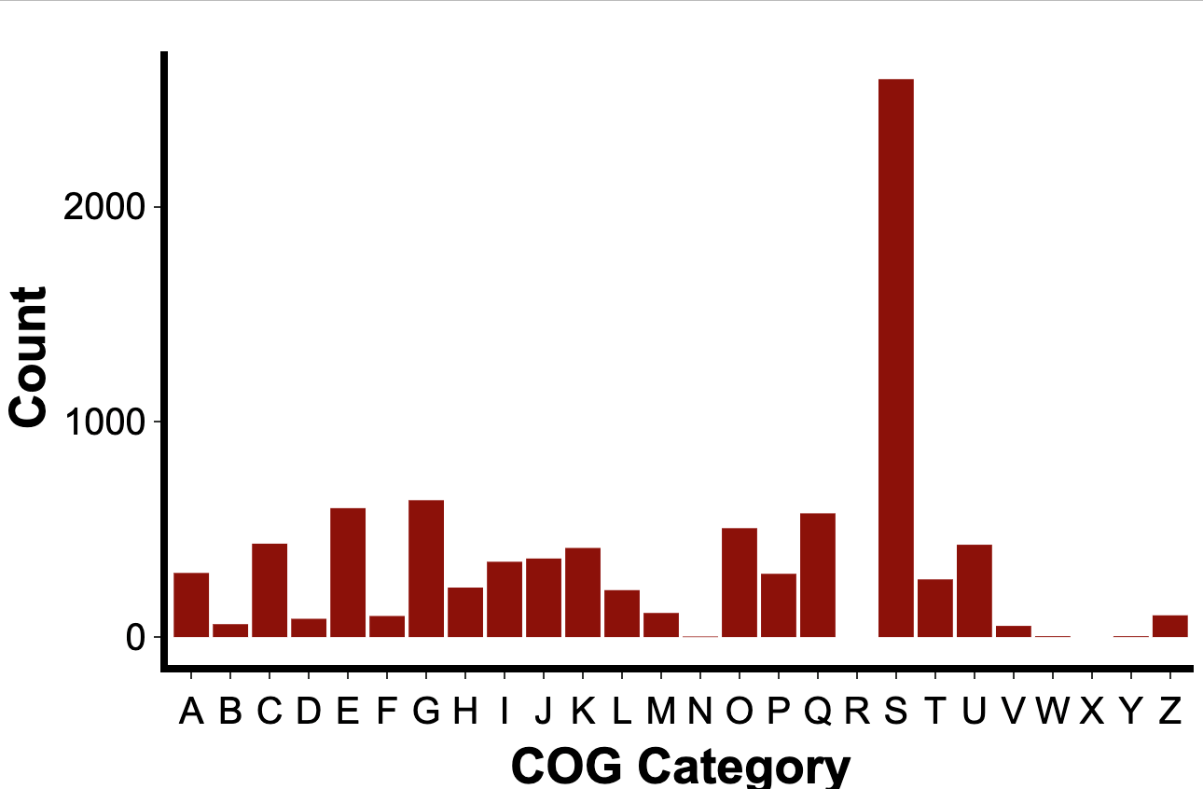


Supplementary Figure 3. Distribution of predicted proteins in *Aspergillus wentii* CBS 141173 across the different COG categories. A: RNA processing and modification. B: Chromatin structure and dynamics. C: Energy production and conversion. D: Cell cycle control, cell division, chromosome partitioning. E: Amino acid transport and metabolism. F: Nucleotide transport and metabolism. G: Carbohydrate transport and metabolism. H: Coenzyme transport and metabolism. I: Lipid transport and metabolism. J: Translation, ribosomal structure. and biogenesis. K: Transcription. L: Replication, recombination. and repair. M: Cell wall/membrane/envelope biogenesis. N: Cell motility. O: Posttranslational modification, protein turnover, chaperones. P: Inorganic ion transport and metabolism. Q: Secondary metabolites biosynthesis, transport. and catabolism. S: Function unknown. T: Signal transduction mechanisms. U: Intracellular trafficking, secretion, and vesicular transport. V: Defense mechanisms. W: Extracellular structures. Y: Nuclear structure. Z: Cytoskeleton. The following returned no hits: R: General function prediction only; X: Mobilome- prophages, transposons.

# Supplementary Table 3: BUSCO assessment of the annotated genome and predicted proteome and transcriptome of *A. wentii* CBS 141173 using the Eurotiales odb10 database.

| **Parameters** | **Genome** | **Protein** | **Transcriptome** |
| --- | --- | --- | --- |
| **Percentage BUSCO** | 94.8% | 95.3% | 94.8% |
| **Complete BUSCO’s** | 3973 | 3992 | 3973 |
| **Complete and single copy BUSCO’s** | 3960 | 3751 | 3960 |
| **Complete and duplicated BUSCOs** | 13 | 241 | 13 |
| **Fragmented BUSCOs** | 27 | 120 | 27 |
| **Missing BUSCOs** | 191 | 79 | 191 |
| **Total BUSCO groups searched** | 4191 | 4191 | 4191 |

**
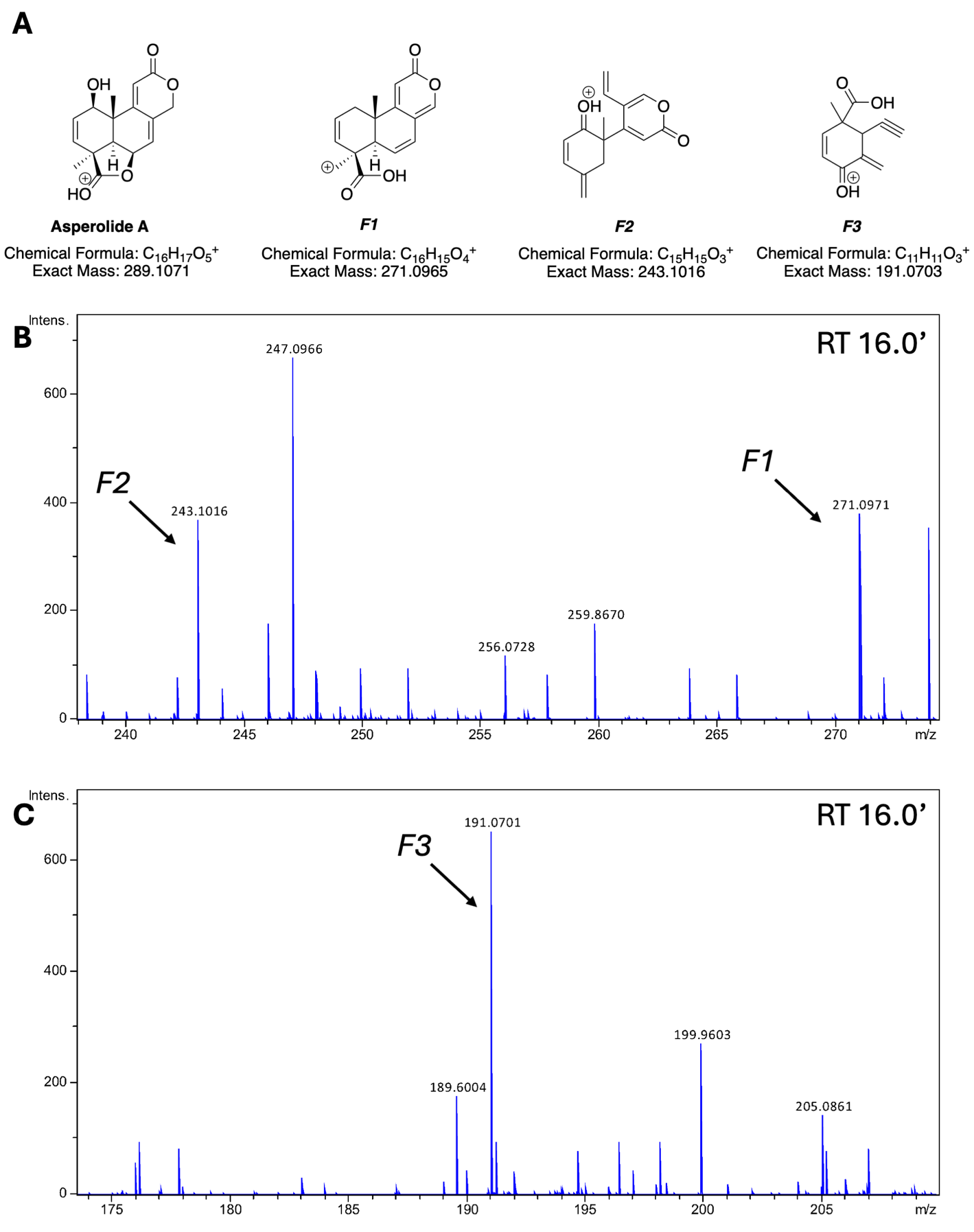
**Supplementary Figure 4. MS^2^ spectra of asperolide A. **A**) Structures of asperolide A and proposed fragments derived from it in MS^2^. **B**) MS^2^ spectrum showing species *F1* and *F2*; *m/z* expected for *F1* = 271.0965; *m/z* expected for *F2* = 243.1016. **C**) MS^2^ spectrum showing species *F3*; *m/z* expected for *F3* = 191.0703;

# Supplementary Table 4: Transcriptomic hitcounts of terpene synthase genes in *Aspergillus wentii* CBS 141173 at two timepoints (7 and 30 days) performed in duplicate (A and B), and compared to the housekeeping gene beta-tubulin.

Genes highlighted in bold (AwTS3, -5, -9, and -10) show the highest expression across the samples analysed.

| Terpene synthase | Gene ID | 7A | 7B | 30A | 30B |
| --- | --- | --- | --- | --- | --- |
| AwTS1 | AWENTII_000319 | 99 | 164 | 28 | 42 |
| AwTS2 | AWENTII_001688 | 1 | 0 | 8 | 4 |
| AwTS3 | **AWENTII_002622** | **399** | **596** | **573** | **589** |
| AwTS4 | AWENTII_003014 | 229 | 184 | 4 | 0 |
| AwTS5 | **AWENTII_006486** | **662** | **765** | **529** | **602** |
| AwTS6 | AWENTII_006746 | 44 | 48 | 49 | 59 |
| AwTS7 | AWENTII_009676 | 16 | 31 | 116 | 131 |
| AwTS8 | AWENTII_009792 | 96 | 164 | 0 | 2 |
| AwTS9 | **AWENTII_010168** | **479** | **474** | **1249** | **1248** |
| AwTS10 | **AWENTII_012038** | **1270** | **1420** | **1998** | **2089** |
| AwTS11 | AWENTII_000061 | 0 | 0 | 3 | 5 |
| AwTS12 | AWENTII_005314 | 1 | 0 | 0 | 0 |
| AwTS13 | AWENTII_005350 | 11 | 7 | 21 | 7 |
| AwTS14 | AWENTII_006388 | 33 | 41 | 11 | 16 |
| AwTS15  AwTS16 | AWENTII_006883  AWENTII_011959 | 117  7 | 240  1 | 111  25 | 161  29 |
| Beta-tubulin | AWENTII_000951 | 6369 | 6465 | 2267 | 2106 |

# Supplementary Table 5: NCBI BioProject ID submission and Accession number of *A. wentii* CBS 141173.

| SUBID | BioProject | BioSample | Localid | Accession |  |
| --- | --- | --- | --- | --- | --- |
| SUB14389936 | PRJNA1133277 | SAMN42381744 | scaffold_1 | CP165651 |  |
| SUB14389936 | PRJNA1133277 | SAMN42381744 | scaffold_2 | CP165652 |  |
| SUB14389936 | PRJNA1133277 | SAMN42381744 | scaffold_3 | CP165653 |  |
| SUB14389936 | PRJNA1133277 | SAMN42381744 | scaffold_4 | CP165654 |  |
| SUB14389936 | PRJNA1133277 | SAMN42381744 | scaffold_5 | CP165655 |  |
| SUB14389936 | PRJNA1133277 | SAMN42381744 | scaffold_6 | CP165656 |  |
| SUB14389936 | PRJNA1133277 | SAMN42381744 | scaffold_7 | CP165657 |  |
| SUB14389936 | PRJNA1133277 | SAMN42381744 | scaffold_8 | CP165658 |  |
